# Editorial: Science diplomacy and neocolonialism: lessons from the field with a view to the future

**DOI:** 10.3389/fpubh.2025.1700272

**Published:** 2025-10-09

**Authors:** Rasha Bayoumi, Tania Bosqui, Abdullah Awad, Rana Dajani

**Affiliations:** ^1^University of Birmingham, Dubai, United Arab Emirates; ^2^American University of Beirut, Beirut, Lebanon; ^3^University of Cambridge Public Health, Cambridge, United Kingdom; ^4^The Hashemite University, Az-Zarqa, Jordan

**Keywords:** science diplomacy, public health, global south, equity, governance, capacity building, strategic partnerships

## Introduction

Science diplomacy, understood as the use of scientific cooperation to inform foreign policy and advance shared public goods, has long unfolded under neocolonial conditions that marginalize researchers and publics in the Global South (1). Power asymmetries shape what questions are asked, who leads, how evidence is valued, and who ultimately benefits (1). Recent crises, from COVID-19 to protracted conflicts including those in Ukraine and Palestine, have further exposed how public health collaboration can reproduce inequities rather than redress them (Dajani et al.). Yet across the Global South, practitioners are assembling more inclusive and flexible models that include co-governed research, regionally anchored training platforms, culturally validated metrics, and financing arrangements designed to protect local leadership. This Research Topic examines how public health research and promotion interact with science diplomacy in such contexts, identifies practical strategies for multilateral and interdisciplinary partnerships, and showcases case studies that challenge hierarchies and travel across diverse settings (see included articles in Table 1). While prior work has often centered South America and Asia, this Research Topic highlights under-studied Middle East and African contexts, contributing to a more balanced comparative literature on science diplomacy.

**Table 1:** Mapping the four key themes to the relevant Research Topic articles.

**Theme**	**Articles**
Governance and policy frameworks: from control to collaboration	• Dajani et al.; Neocolonialism and science diplomacy: lessons learned from the field • Dabbagh; Reflections on mental health policy and practice in the Middle East • Adam et al.; Decolonizing global health research: experiences from the women in health and their economic, equity and livelihood statuses during emergency preparedness and response (WHEELER) study • Oyugi et al.; The regional training centre for the emergency medical teams initiative in the WHO African region: a review of the development and progress over the past 4 years • Al Khatib et al.; Mitigating climate change impacts on health: a comparative analysis of strategies in Saudi Arabia and Lebanon • Bayoumi et al.; Enhancing cultural sensitivity in the implementation of the Fertility Quality of Life Tool in Sudan: a science diplomacy perspective • Murphy et al.; The long shadow of accumulating adverse childhood experiences on mental health in the United Arab Emirates: implications for policy and practice
Capacity building and knowledge exchange: grounding global health in local realities	• Othman and Selnow; Community health workers: a narrative review of a curriculum and training program for low-income communities facing limited access to healthcare • Feng et al.; The effects of traditional Chinese exercises on anxiety and depression in adults: a systematic review and network meta-analysis • Adam et al.; Decolonizing global health research: experiences from the women in health and their economic, equity and livelihood statuses during emergency preparedness and response (WHEELER) study • Millar et al.; Building capacity and capability for science diplomacy: challenges in decolonizing the curriculum for Global Health System Leadership • Rockowitz et al.; Fostering cultural resilience: assessing the success of a locally engaged and adapted mental health intervention in Gaza • Salim; The burden of trauma in the life of a refugee
Strategic partnerships and resource mobilization: restructuring power and participation	• Oyugi et al.; The regional training centre for the emergency medical teams initiative in the WHO African region: a review of the development and progress over the past 4 years • Al Khatib et al.; Mitigating climate change impacts on health: a comparative analysis of strategies in Saudi Arabia and Lebanon • Dajani et al.; Volunteer programs and empowerment in Jordan • Bayoumi et al.; Enhancing cultural sensitivity in the implementation of the Fertility Quality of Life Tool in Sudan: a science diplomacy perspective • Adam et al.; Decolonizing global health research: experiences from the women in health and their economic, equity and livelihood statuses during emergency preparedness and response (WHEELER) study • Dajani et al.; Neocolonialism and science diplomacy: lessons from the field
Design, implementation, and impact assessment: operationalizing equity and accountability	• Dajani et al.; Volunteer programs and empowerment in Jordan • Bayoumi et al.; Enhancing cultural sensitivity in the implementation of the Fertility Quality of Life Tool in Sudan: a science diplomacy perspective • Murphy et al.; The long shadow of accumulating adverse childhood experiences on mental health in the United Arab Emirates: implications for policy and practice • Rockowitz et al.; Fostering cultural resilience: assessing the success of a locally engaged and adapted mental health intervention in Gaza • Feng et al.; The effects of traditional Chinese exercises on anxiety and depression in adults: a systematic review and network meta-analysis • Tsega et al.; Socioeconomic inequality in financial hardship in accessing quality healthcare services in Ethiopia: a community-based cross-sectional study • Salim; The burden of trauma in the life of a refugee

## Governance and policy frameworks: from control to collaboration

Science diplomacy is recast in these contributions as a governance project, not only about brokering ties across borders but about redesigning the rules, incentives, and relationships through which knowledge is produced and applied. The common pivot is away from control, top-down, externally defined, and often Western-led, toward collaboration grounded in reciprocity, plural knowledge systems, and local ownership. The WHEELER study in Kenya exemplifies this turn by embedding Community Research Advisory Groups and Local Advisory Boards into the research process and applying gender analysis to ensure representation (Adam et al.). The WHO EMT Training Center in Addis Ababa illustrates another model of collaborative governance, transforming an evacuation site into a regional training hub aligned with the African Union's Agenda 2063, while candidly acknowledging gaps in monitoring and sustainability (Oyugi et al.). Reflections on public mental health in Palestine and the UAE underscore how governance can emerge from daily practice rather than imported models, with narrative approaches to suicide and autism pathways designed through community consultation (Dabbagh). Critiques of ethics rule-setting bodies reveal how the absence of Muslim scientists undermines legitimacy, while the collapse of the UK's Global Challenges Research Fund highlights how dependence on short-cycle, externally controlled grants unravels equity projects (Dajani et al.). Collectively, these studies argue that equitable governance requires specifying co-decision structures from proposal to publication, reforming ethics and standard-setting bodies for cultural and regional diversity, investing in regional capacity platforms with robust monitoring and evaluation, and designing multi-year funding instruments with safeguards against political or budgetary shocks. These cases also demonstrate Global South researchers as protagonists, shaping agendas beyond state hierarchies and asserting agency in international systems.

## Capacity building and knowledge exchange: grounding global health in local realities

Capacity building is presented here not as a one-way transfer of “best practices” but as a dialogic, context-rooted process that enables communities and researchers to co-produce priorities and solutions. The Community Health Worker program developed by WiRED International reframes workforce expansion as community-owned professionalization, offering open, offline-capable curricula for rapid and low-cost training that can be adapted to local languages and norms (Othman and Selnow). A meta-analysis of Traditional Chinese Exercises shows that cultural consonance is not an accessory but a mechanism of effectiveness, since embedded practices such as tai chi and qigong drive adherence and outcomes, raising questions about how such modalities can be sensitively adapted while maintaining therapeutic cores (Feng et al.). WHEELER again provides lessons on reciprocity by design, combining local resource centers, mentorship ladders, and sense-making workshops into a learning system that endures beyond a single project (Adam et al.). An article on international branch campuses highlights the paradoxes of decolonizing education from privileged institutional settings but demonstrates how dialogic pedagogy and co-developed competency frameworks with regional partners can make education itself a form of diplomacy (Millar et al.). Finally, the Tarkiz program in Gaza illustrates long-horizon adaptation, showing how 15 years of locally stewarded mental health interventions anchored in community participation can sustain impact under chronic crisis (Rockowitz et al.). Taken together, these contributions argue that capacity building is most effective when it is open, dialogic, culturally embedded, and infrastructural, producing two-way change in which both academic teams and community partners gain new capabilities.

## Strategic partnerships and resource mobilization: restructuring power and participation

Effective science diplomacy also depends on who convenes and who controls resources. Several studies highlight models that de-center extractive funding and institutionalize shared leadership. The EMT hub in Addis Ababa represents a sustained, multi-actor training platform rooted in the region (Oyugi et al.). A comparative analysis of climate–health strategies in Saudi Arabia and Lebanon demonstrates how different political economies and funding ecosystems shape adaptation priorities (Al Khatib et al.). The Jordan “We Love Reading” initiative shows how participatory systems mapping can reveal empowerment pathways among refugees and host communities, demonstrating that community–academy partnerships can inform public policy (Dajani et al.). Research on infertility in Sudan demonstrates how adapting tools such as FertiQoL to cultural realities strengthens the case for investment in infertility care, linking measurement choices to resource mobilization (Bayoumi et al.). The perspective piece by Dajani et al. broadens this argument, calling for grassroots-driven and reflexive partnerships that resist tokenistic collaborations (Dajani et al.). Together, these contributions suggest that equitable diplomacy requires multi-year partnerships with co-leadership clauses, financing tied to community-defined metrics, and investment in platforms such as regional hubs rather than isolated projects.

## Design, implementation, and impact assessment: operationalizing equity and accountability

Assessment frameworks are most meaningful when they measure what communities value. Several articles illustrate how to embed accountability and equity into design and evaluation. A participatory systems mapping study in Jordan used Fuzzy Cognitive Mapping with Syrian refugees and Jordanian women to generate causal models of empowerment that are owned by the community and can be simulated for policy scenarios (Dajani et al.). Research on FertiQoL in Sudan shows that cognitive interviewing and cultural adaptation challenge universalizing measures that miss local lived experience (Bayoumi et al.). A study from Ethiopia quantifies socioeconomic gradients in access to quality care (Tsega et al.), while population-level research in the UAE links cumulative childhood adversity to adult mental health, highlighting the importance of early identification and trauma-informed systems (Murphy et al.). The Tarkiz program in Gaza provides an example of community-defined success criteria evolving over 15 years (Rockowitz et al.), while a network meta-analysis of Traditional Chinese Exercises illustrates how evidence synthesis can elevate non-Western practices into global discourse (Feng et al.). Across these cases, the message is clear: equity requires co-created theories of change, cultural validation protocols before cross-setting use of measures, and transparent reporting of authorship balance, principal investigator location, and data stewardship. It also requires closing practice-to-policy loops so that feedback from service delivery informs curricula and national guidelines. Future work should collate such models dynamically, producing flexible frameworks that allow lessons learned in one context to be adapted across others.

## Conclusion

This Research Topic advances a pragmatic agenda for equitable science diplomacy, emphasizing representative governance, reciprocal capacity ecosystems, co-led and shock-resilient financing, and equity-first impact assessment. Across diverse contexts, the studies demonstrate that shifting from extractive ties to shared authority is feasible when participation is specified, cultural validity is designed in, and resources are stabilized. Looking forward, three priorities stand out: funders should require co-governance structures and equity audits, ministries should invest in regional platforms that link training to service change, and journals and consortia should adopt standardized equity metrics in reporting. Done this way, science diplomacy becomes not an instrument of soft power but a practice of shared power in service of global public health equity.
